# Mitochondria as a target in cancer treatment

**DOI:** 10.1002/mco2.16

**Published:** 2020-07-15

**Authors:** Yu'e Liu, Yufeng Shi

**Affiliations:** ^1^ Tongji University Cancer Center Shanghai Tenth People's Hospital of Tongji University School of Medicine Tongji University Shanghai China; ^2^ Center for Brain and Spinal Cord Research School of Medicine Tongji University Shanghai China

## Abstract

Mitochondria are biosynthetic, bioenergetic, and signaling organelles existing in almost all eukaryotic cells, and their dysregulated function has been proved to be essential for tumorigenesis, tumor development, and tumor metastasis. In this short review, first, we briefly summarize the historic misunderstanding of mitochondria in tumors, and then come up with a current view that mitochondria play a pivotal role in tumor cells; second, we review how tumor cells rewind mitochondrial function for their oncogenic purpose via known or unknown mechanisms by key oncogenes or tumor suppressors; third, we go through reagents and strategies currently available targeting mitochondria when treating tumors. Recently, merging data suggest that slow cycling cancer cells/cancer stem cells have distinctive mitochondrial metabolism comparing to bulk tumor cells and mitochondria inhibitors seem to be promising to target them, which are resistant to traditional radio and chemotherapies. We thus discuss role of mitochondria in these cancer stem cells and summarize mitochondria as a target from different aspects.

## Graphical abstract

Oncoproteins or tumor suppressors reprogram mitochondrial function for tumorigenesis and tumor maintenance. Multiple mitochondria targeting reagents have been developed to treating tumors.

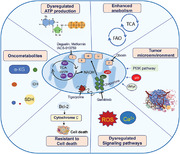

## 1 HISTORIC VIEW OF MITOCHONDRIA IN CANCER

The importance of mitochondria in cancer has been ignored for a long time. Around 100 years ago, Otto Warburg discovered that cancer cells undergo aerobic glycolysis even in the presence of oxygen, which is referred as the “Warburg effect.” As the “Warburg effect” is observed in a wide range of cancer cells, Warburg further reasoned that cancer might arise from impaired mitochondria.^1^, ^2^ In fact, as most nonproliferating, differentiated cells mainly depend on the efficiency of ATP production through oxidative phosphorylation (OXPHOS) to maintain their integrity, aerobic glycolysis–based F‐18‐fluorodeoxyglucose positron emission tomography “PET” scan is currently the most widely used tumor‐detecting technology. However, now we know although damaged mitochondria can render the Warburg effect in cells, most cancer cells undergo aerobic glycolysis with their mitochondria remaining intact, which can be seen in both cultured tumor cells and tumor cells in patients with technologies (such as ^13^C Glucose tracing) applied recently.^3^

**FIGURE 1:**
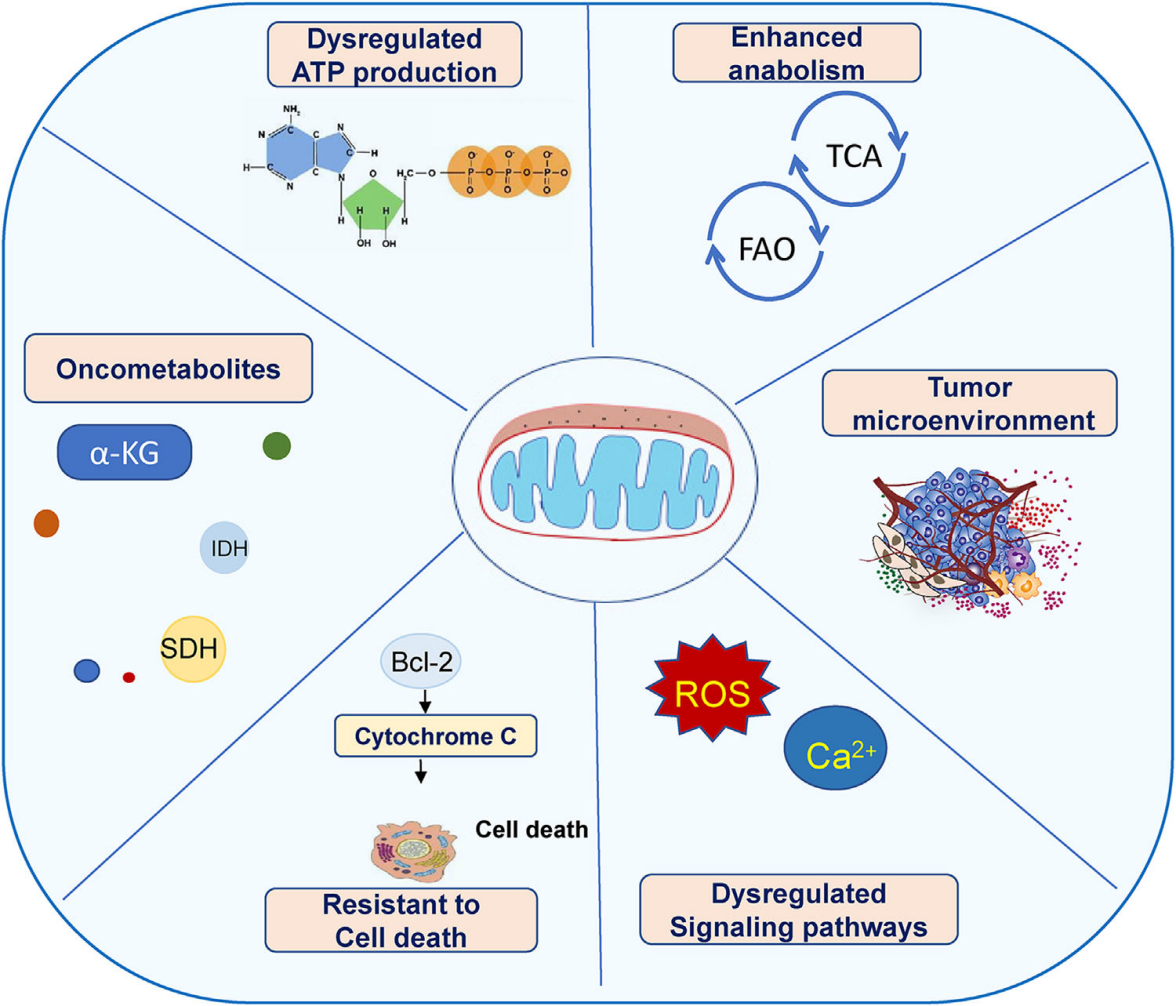
Role of mitochondria in tumor. Role of mitochondria in bioenergetics, cell death, biogenesis, signaling, and tumor microenvironment

So far, the importance of mitochondria in cancer has been proved experimentally in many ways. It is found that inhibition of mitochondria by inactivation of a mitochondrial transcription factor (Tfam) or poisoning mtDNA (*p*0 cells) compromises tumorigenesis.^4^, ^5^. Of note in these settings, growth of mtDNA‐depleted *p*0 tumors is associated with the incorporation of host tissue mitochondrial genomes and restoration of mitochondrial function. In addition, evidences show mitochondrial metabolism and mitochondrial reactive oxygen species (ROS) generation are essential for Kirsten rat sarcoma viral oncogene homolog (KRAS)‐driven tumorigenicity,^5^ and mitochondria OXPHOS inhibition by small molecules targeting OXPHOS complex I and complex V induces tumor cell death and reduces tumor growth in animal models.^6^, ^7^, ^8^ Further, data from a recent clinical trial shows suppression of OXPHOS via a combination treatment of venetoclax and azzcytidine induces leukemia stem cell death and results promising clinical outcome.^9^, ^10^ These evidences from the mouse tumor model and cancer patient are opposite to what Warburg envisioned and demonstrate critical roles of mitochondria in tumor initiation, maintenance, and growth (Figure 1).

## 2 ROLES OF MITOCHONDRIA IN CANCER

### 2.1 Mitochondria in energy metabolism

A major function of mitochondria is to produce ATP by its OXPHOS pathway, which is coupled with tricarboxylic acid cycle (TCA) and fatty acid oxidation (FAO) pathways. Although tumor cells can use glycolysis to supply ATP, mitochondrial ATP production is more efficient and allows broader substrates, enabling tumor cells to conduct an extensive and high plastic metabolic rewiring and survive in otherwise harsh nutrient conditions. Tumor consumes large amounts of glucose, but glucose concentration could be low for tumor cells in certain area; and these tumor cells use other carbon sources instead (including but not limited to acetate, lactate, serine, and glycine), which normally require a fully functional mitochondria to process for ATP production.^11^, ^12^, ^13^ In addition, mitochondria seem to be the main source for ATP production when tumors are under certain scenario. For example, in a KRAS‐driven pancreatic ductal adenocarcinoma (PDAC) mouse model, oncogenic KRAS blockage cause massive tumor cell death. The remaining tumor cells are highly sensitive to OXPHOS inhibition, and OXPHOS complex V (ATP synthase) inhibition by Oligomycin A eliminates remaining tumor cells and greatly reduces PDAC relapse.^8^, ^14^

### 2.2 Mitochondria in biomass synthesis

As a central metabolic organelle, mitochondria are critical for providing intermediates required for biomass synthesis, which includes fatty acids, amino acids, and nucleotides, required as building blocks for cancer cell growth. In this regard, one essential role of the mitochondrial electron transport chain (ETC) is enabling aspartase synthesis in proliferating cancer cells, and ETC inhibition greatly reduces cancer cell growth, which can be partially rescued by supplementation of aspartase in medium.^15^ ETC activity is coupled with pyrimidine synthesis too. Dihydroorotate dehydrogenase (DHODH), a critical enzyme required for de novo pyrimidine synthesis, is located on the inner mitochondrial membrane, where it oxidizes dihydroorotate to orotate. Unlike other dehydrogenases using NAD+ or NADP+ as electron acceptor, DHODH transfers electrons to ubiquinone, substrates for the ETC complex III, and thus links nucleotide synthesis with mitochondrial energy metabolism.^16^ The importance of mitochondrial contribution of building blocks for cancer cell growth has also been demonstrated as activity of several mitochondria enzymes/proteins involved in synthesis of fatty acids, amino acids, and nucleotides is upregulated in multiple different tumors. Mitochondrial proline synthesis is critically important for tumor cell growth. Enzymes required for proline biosynthesis, mitochondrial NAD(P)H‐dependent enzyme pyrroline‐5‐carboxylates reductase, and P5C biosynthetic enzyme delta‐1‐pyrooline‐5‐carboxylate synthase (P5CS), are found to be increased in cancers, such as prostate, lymphoma, and other types of tumors.^14^, ^17^, ^18^, ^19^

### 2.3 Mitochondria, ROS, and calcium storage

Mitochondria are major organelles for ROS generation, redox molecules generation, and calcium storage; therefore, mitochondria function as a regulator for multiple related signaling, which is essential for tumor cells to cope with surrounding environment.^20^ ROS is mainly generated inside mitochondria by OXPHOS complexes and can react with DNA, proteins, and lipids. At physiological levels, ROS functions as “redox messengers in intracellular signaling and promote cancer cell growth, whereas excess ROS induces protein damage, leads to the integrated stress response of mitochondria and enables the DNA or other molecules release from mitochondria to cytoplasm, activates the DNA effector or cell death pathway, and ultimately causes autoimmunity or triggers cancer cell death,^21^ thus ROS directs the autoimmunity, life, or death and have to be tightly regulated in cancer cells. Mitochondria are also the main sites of calcium storage, which control intracellular Ca^2+^ signaling, cell metabolism, cell survival, and so on.^22^ Increased mitochondrial Ca^2+^ may trigger cell death by necrosis or cell death related with sustained opening of mitochondrial permeability transition pore (mPTP).^23^ Interestingly, mPTP was found to be inactivated in most of cancer cells, which likely is a mechanism for cancer cell survival in unfavorable conditions.^24^

### 2.4 Mitochondria and programmed cell death

Mitochondria play an essential role in programmed cell death (Figure 1),^25^ mediate the intrinsic apoptosis program characterized by cytochrome *c* release, which is regulated by Bcl‐2 family proteins governing the mitochondrial outer membrane permeabilization (MOMP). Bcl‐2 family proteins can be divided into members that function as preventing apoptosis (prosurvival) and those that induce apoptosis (proapoptotic) operating as a core to integrate stress‐signaling networks. It is now known that these Bcl‐2 proteins are often dysregulated in many cancers often with prosurvival members highly expressed or proapoptotic members downregulated, rendering increased survival of cancer cells.^26^, ^27^ Recently, Llambi et al identified a noncanonical Bcl‐2 family effector, BCL‐2 ovarian killer (BOK). BOK activity is regulated by proteasomal degradation, and it induces mitochondrial apoptosis in the absence of BAX and BAK and promotes apoptosis independent of other BCL‐2 family proteins.^28^ So far, no reports show a role of BOK in cancers, but it will be very interesting to see whether BOK activity is dysregulated in certain types of cancers. The cell death related MOMP can also be regulated by other mitochondrial outer membrane proteins, such as voltage‐dependent anion channel VDAC2. In certain tumor cells, the tumor suppressor lipids ceramides bind VDAC2 to trigger mitochondrial apoptosis.^29^

### 2.5 Mitochondria and stem cells

It is now appreciated that mitochondria play a pivotal role in stem cell maintenance in normal tissues.^30^ Multiple aspects of mitochondria function, such as mitochondrial metabolism, dynamics, and signaling pathways determine/influence stem cell identity, self‐renewal, and fate decisions (see the recent excellent review in Ref. ^31^). Cancer stem cells (CSCs) is a term that is borrowed from stem cells in normal tissues and referred to a subpopulation of high stemness and high tumorigenic tumor cells, which can regenerate the whole tumor after treatment.^32^, ^33^, ^34^, ^35^, ^36^, ^37^ CSCs have been identified in multiple tumors, like tumors of hematopoietic system, breast, prostate, pancreas, colon, skin, and brain.^32^, ^33^, ^34^, ^35^, ^38^, ^39^ Due to their stemness, relative quiescence, and multiple drug resistance, these slow‐cycling CSCs are often responsible for tumor metastasis, treatment resistance, and relapse, common features for many different tumors independent of organ of origin. Recently, emerging evidences show CSCs in certain types of tumors have distinct metabolism comparing to normal wild‐type cells or bulk tumor cells and highly rely on mitochondria for survival.^7^, ^9^, ^10^ Mitochondria‐targeting reagents could eliminate these CSCs and prolong survival by its own or in combination with other drugs.^7^, ^9^, ^10^

## 3 MITOCHONDRIA REPROGRAM IN CANCER

**FIGURE 2:**
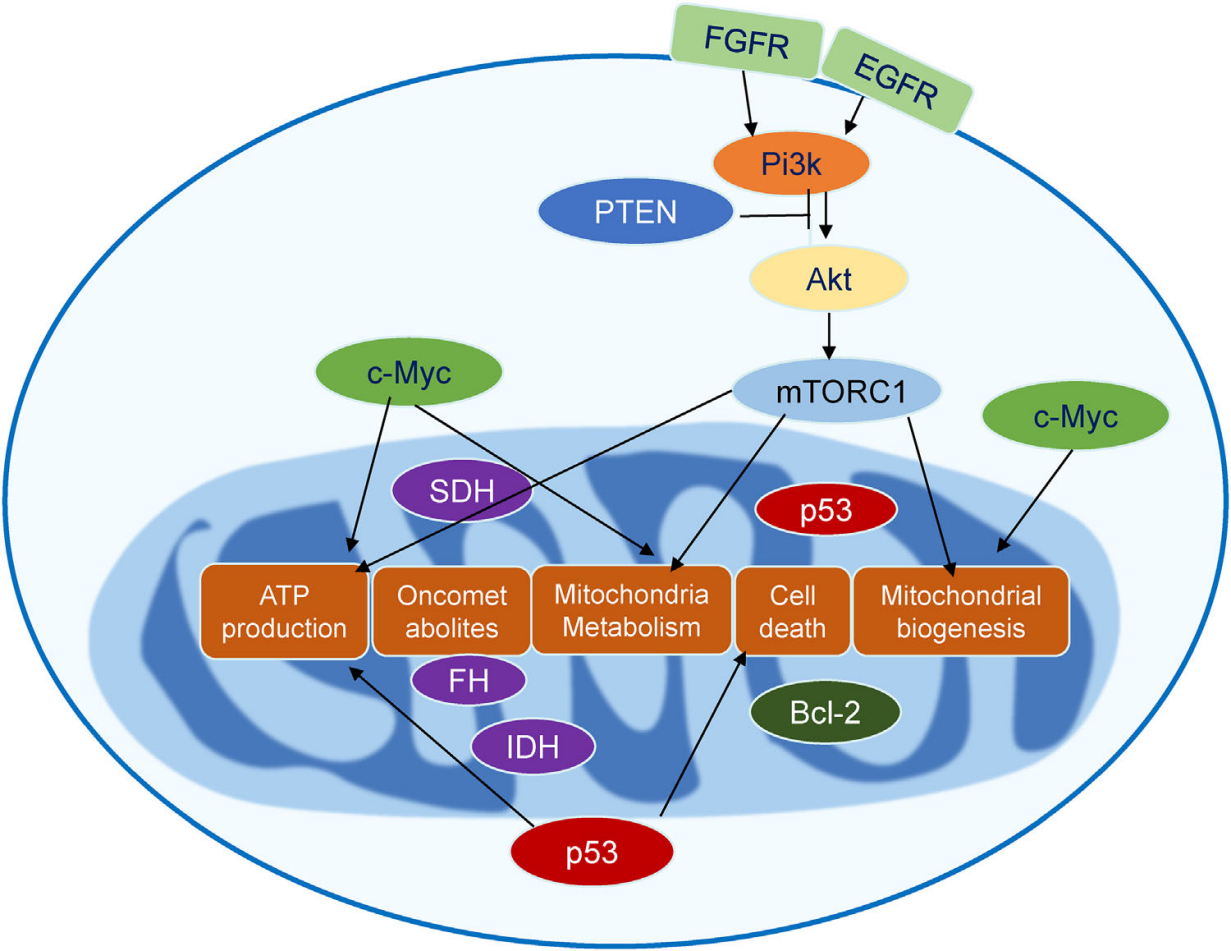
Mitochondria reprogramming in tumor. Some oncoproteins or tumor suppressors function within mitochondria (such as IDH1/2, SDH, and FHs) generating oncogenic metabolites initiating tumor formation, and many others directly or indirectly affect mitochondrial function (mutations including but not limited to that in Pi3k/Akt /mTOR pathway, *Tp*53, and *Myc*) and reprograming mitochondria metabolism enabling tumor transformation

Accumulation of mutations in oncogenes or tumor suppressors in somatic cells leads to the transformation of normal cells into malignant tumor cells. Some oncoproteins or tumor suppressors function within mitochondria (such as IDH1,^40^ SDH, and FHs^41^) generating oncogenic metabolites needed for tumor initiation (see a recent great review in Ref. ^42^), and many others directly or indirectly affect mitochondrial function (mutations including but not limited to that in the Pi3k/Akt pathway,^43^ Tp53,^44^ and Myc^45^) and reprograming mitochondria metabolism enabling tumor transformation. Research in the past decades shows that mitochondria reprogramming plays an essential role in tumor initiation and maintenance (Figure 2). Thus, understanding how oncogenes/tumor suppressors altering mitochondrial metabolism will be critical for understanding tumor initiation and evolution, and developing tools and strategies to precisely and effectively target these organelles when treating tumors.

### 3.1 Mitochondria and oncometabolites

Oncometabolites refer to metabolites which are remarkably increased in tumors due to certain mutations and play oncogenic roles. So far only few metabolites (2D‐HG, succinate, and fumarate) are considered as oncometabolites, which aroused from mutations in nuclear‐encoded mitochondria enzymes (IDH1, SDH, and FH, respectively). It is believed that oncometabolites can assist in reprogrammed enzymatic pathways, which play tumorigenic roles. Small molecules have been developed for treating tumors carrying mutated enzymes responsible for the accumulated oncometabolite.

Isocitrate dehydrogenase 1 and 2 (IDH1 and IDH2) are two of the most frequently mutated metabolic genes in human cancer. IDHs are metabolic enzymes catalyzing the oxidative decarboxylation of isocitrate to α‐ketoglutarate (α‐KG), NAD(P)H, and CO_2_ and epigenetically control gene expression. Mutation in an IDH enzyme in cancer was first identified in colon cancer. It was subsequently discovered in glioma, acute myelogenous leukemias (AMLs), and other types of tumors.^40^, ^46^ Somatic point mutations in IDH1/2 is a gain‐of‐function mutation, resulting in the accumulation of an oncometabolite, the D‐2‐hydrocyglutarate (D‐2HG).^47^ D‐2HG functions as a competitive inhibitor for α‐KG‐dependent epigenetic regulators, such as the ten‐eleven translocation (TET) family of 5‐methylcytosine hydroxylases and Jumonji‐C domain‐containing histone demethylases, both of which alter epigenetic state of a cell genome, leading to dysregulated gene expression and contributing transformation of normal wild‐type cells into tumor cells.

Succinate dehydrogenase (SDH) and fumarate hydratase (FH) function sequentially in the TCA cycle and are found in familial cancer syndromes with loss of one allele as somatic mutation and loss of both alleles in tumors.^48^, ^49^ All TCA cycle intermediates can be found throughout the body, but some tumors have extreme levels of succinate and/or fumarate resulted from loss of function for SDH and/or FH, respectively. Although succinate and fumarate can be seen as oncometabolites, their role in cancer is likely to be related to their nonmetabolic functions^50^ and through epigenetic regulation of gene expression. However, the mechanisms underpinning the link between metabolic dysregulation and cancer initiation remain only partially understood.

### 3.2 Mitochondrial reprogramming by the oncogenic PI3K/Akt/mTOR pathway

PI3K/Akt and the mammalian target of rapamycin (mTOR) signaling pathway is crucial to cell growth, cell metabolism, and survival and is the most frequently dysregulated pathway in cancer. The dysregulation of PI3K/Akt/mTOR can take place in many levels including but not limited to (1) gain‐of‐function mutation or amplification of tyrosine kinase receptor, for example, EGFR, FGFR, and so on; (2) mutations in signaling kinases, for example, *Braf*, *Kras*,and so on; (3) loss/mutation of the *Pten*/*Nf1* tumor suppressors, key phosphatase that shut off this pathway. Activation of the PI3K/Akt pathway directly promotes glucose carbon flux into biosynthetic pathways, upregulates mitochondrial anabolic metabolism, and reprogramming of mitochondrial citrate metabolism, all of which need a functional mitochondrion and contribute to transforming a normal wild‐type cells into tumor cells.^51^ The mTOR complexes as the downstream effectors for the PI3K/Akt pathway integrates growth signaling and nutrient‐regulating translation, anabolic metabolism, and autophagy.^52^ mTORC1 not only affects mitochondrial biogenesis but also stimulates multiple mitochondrial metabolic pathways. For example, mTORC1 can inhibit SIRT4 to activate glutamate dehydrogenase (GDH) and then upregulate glutaminolysis.^53^ mTORC1 promotes protein synthesis and mitochondrial metabolism; it can also induce the mitochondria folate pathway to upregulate purine synthesis and promote tumor cell growth.^54^

### 3.3 Mitochondrial reprogramming by tumor suppressor Tp53

More than 50% tumors have loss‐of‐function mutant in *Tp53* gene. The classical role of Tp53 as tumor suppressor is its transcription regulation of cell cycle and apoptotic genes. It is recently appreciated that the tumor suppressor function of Tp53 is also reached via regulation of genes involved in cellular metabolism, demonstrating a functional role for mutant Tp53 in cancer metabolism. Tp53 or its mutation regulates multiple nuclear and mitochondria proteins through transcriptional regulation or protein modification to affect mitochondrial biogenesis and mitochondria function. Tp53 inhibits glycolysis but promotes transcription of genes involved in mitochondria oxidative phosphorylation and fatty acid oxidation in cancer cells.^55^ Recent data further shows that α‐KG, one of the key products of the TCA cycle, is an effector of Tp53‐mediated tumor suppression. The accumulation of α‐KG in p53‐deficient tumors can drive tumor cell differentiation and antagonize malignant progression.^56^ An increases in the level of α‐KG by the suppression of Oxoglutarate Dehydrogenase (OGDH) is sufficient to impose a Tp53‐like chromatin and transcriptional profile in tumor cells that lack Tp53, which enables cells to reacquire a premalignant identity.^56^ In addition, Tp53 directly function as regulator in cell death related pathways, Bcl‐2 family regulated the cell apoptotic pathway and mitochondria permeability pore related cell death.^57^, ^58^, ^59^ Thus, oncogenic mitochondrial regulation by Tp53 is both through its transcription activity and direct regulation for activity of mitochondrial proteins.

### 3.4 Mitochondria reprogramming by oncoprotein Myc

The oncogene *Myc* is deregulated in more than 50% cancers, and its overexpression is often associated with poor prognosis and short survival. The classic tumorigenic function for Myc protein includes its regulation of cell cycle, protein biosynthesis, DNA repair, and signaling transduction regulation ‐and so on. Recently, numerous studies have linked the oncogenic Myc function with reprogramming of mitochondrial metabolism. The importance of mitochondrial regulation in Myc‐driven tumors is first identified in a cDNA screen for genes rescuing cell growth of *c‐Myc*‐null cells. The mitochondrial protein SHMT2, which functions in the first reaction in 1C metabolism, was identified as the only target that could partially rescue the growth of *Myc* deficient cells.^60^ However, Myc‐mediated mitochondrial reprogramming is far beyond that. Myc upregulates the expression of glucose transporter (Glut1) and the enzyme glutaminase (GLS), which enhances glucose and glutamine metabolism, respectively. As a translational activator, Myc competes with SRSF1 and RBM42 and increases expression of mitochondrial respiration chain proteins, which enhance mitochondrial biogenesis.^61^ All these data thus suggest oncoprotein Myc profoundly regulates mitochondrial function, which plays a critical role in Myc‐driven tumors.

### 3.5 Mitochondrial reprogramming by cell state

CSCs are the stem‐like tumor cells, which are relative quiescent in untreated tumors but are able to regenerate the whole organism when bulk tumor cells were eliminated or removed. Recent data show that mitochondria in normal stem cells are different from their differentiated compartments and are critical for normal stem cells maintenance and self‐renewal.^30^ Although the relationship between mitochondria and CSCs is less clear, several laboratories found that CSCs in multiple different tumor types had unique mitochondrial metabolism comparing to differentiated tumor cells and normal wild‐type cells, implying cell state is another layer of regulation for mitochondrial metabolism.^7^ Cancer stem cell theory is first established in tumors in the hematopoietic system, studies shows leukemic stem cells (LSCs) exhibit unique mitochondrial characteristics with increasing reliance on mitochondrial oxidative phosphorylation, and mitochondrial‐targeting reagents preferentially kill LSCs over normal hematopoietic cells.^62^, ^63^, ^64^ Recent data further show the mitochondrial intermembrane assembly (MIA) pathway is elevated in LSCs, inhibiting the MIA pathway or its downstream substrate COX17 reduces LSC viability.^31^, ^65^

## 4 REAGENTS AND STRATEGIES TARGETING MITOCHONDRIA FOR CANCER THERAPY

Dysregulated energy supply is a hall marker for cancer. Although the finding of upregulated glycolysis a century ago blurred the importance of mitochondria function in cancer, recent findings in this field suggests mitochondria not only play an important role in cancer cell viability but also might be essential for tumorigenesis. Thus, there is a urgent interest in pursuing study of mitochondria biology in cancers and targeting this organelle therapeutically. As different mutations in tumors affect the mitochondria function at a different angle, mitochondria targeting reagents and strategies for tumor treatment should be selected individually according to their tumor type, tumor cell states, and tumor mutation status (Table 1).

**TABLE 1:** Reagents targeting mitochondria for cancer therapy

Reagent's name	Mechanism of action	Reference
Enasidenib	IDH2 ‐mutant inhibitor	^71^, ^72^
Ivosidenib	IDH1‐mutant inhibitor	^84^
Venetoclax and azzcytidine	Suppression of oxidative phosphorylation, induces leukemia stem cell death	^9^, ^10^
Rampamycin	mTOR inhibitor, it reduces the metabolic rate, augments differentiation and inhibits tumor formation.	^85^
Galloflavin	LDH inhibitor	^86^
SB‐204990	ATP citrate lyase chemical inhibitor	^87^
MitoTEMPOL	Antioxidant (reducing ROS)	^88^
Metformin	Complex I inhibitor	^69^
Deguelin	Complex I inhibitor	^68^
IACS‐010759	Complex I inhibitor	^70^
Rotenone	Complex I inhibitor	^79^
Oligomycin	Complex V inhibitor	^8^
Gboxin	Complex V inhibitor	^7^
Tigecycline	Mitochondria protein translation inhibitor	^66^
Gamitrinib	OXPHOS assembly inhibitor	^67^

### 4.1 Oncometabolites in mitochondria as a target

The neomorphic production of oncometabolite D‐2HG is essentially a gain of function mutation of IDH1/2 enzymes, which is a promising target for a small molecule inhibitor. Within years after the initial development, IDH inhibitors enasidenib (IDH1‐mutant inhibitor) and enasidenib (IDH2‐mutant inhibitor) were approved by FDA as a first‐in‐class drug for IDH1‐ and IDH2‐mutated AMLs, respectively^71^.^72^ Although, a full estimation of these inhibitor's effect in IDH‐mutated tumors is still not clear. It is reported that treatment of these drugs‐induced durable remission of IDH‐mutated acute myeloid leukemia, with a significant portion of patients develop differentiation syndrome.^73^ So far, no effective reagents have been developed for tumors carrying mutations in SDH and FH, which are responsible for the accumulation of oncometabolites of succinate and fumarate, respectively.

### 4.2 Mitochondrial biomass synthesis as a target

Mitochondria functioning as a center for cellular metabolism provides intermediates critical for synthesis of DNA, protein, and liquid essential for tumor cell growth. In some tumors, certain metabolic liabilities of cancer cells have been translated into effective therapies. Asparaginase is an enzyme that converts the amino acid asparagine to aspartic acid and ammonia. Asparaginase is an essential target for treatment of acute lymphoblastic leukemia (ALL).^74^ Due to the high rates of protein synthesis, ALL cells require a constant supply of aspartic acid, which can be eliminated by systemic administration of asparaginase inhibitors. Serine hydroxy methyltransferase 2 (SHMT2) is a key enzyme in serine/glycine biosynthesis and one‐carbon metabolism. Multiple studies show that SHMT2 plays critical roles in tumor growth and progression in a variety of cancer types, especially in tumors with Myc protein highly expressed. Genetic or pharmacologic inhibition of SHMT2 renders reduced tumor growth or lengthens survival of tumor‐bearing mice.^75^

### 4.3 Mitochondrial signaling as a target

Mitochondria serve as signaling organelles for ROS, calcium, apoptosis, and many others. Reagents have been developed to target some of these mitochondrial function for tumor treatment. Due to hypermetabolism in cancer cells, maintenance of redox homeostasis becomes extremely important. Reagents‐promoting ROS generation or disturbing the redox homeostasis, such as 2‐methoxyestradiol, cisplatin, and buthionine sulfonimine, could induce cancer cell death and inhibit tumor growth. Mitochondria Bcl‐2 family proteins play a major role in tumor cell survival, and antiapoptotic proteins, such as Bcl‐2 or Mcl‐1, are often highly expressed in tumor cells.^76^ Hence, inhibitors specific for Bcl‐2 or Mcl‐1 have been developed as direct inducers for tumor cell apoptosis in chronic lymphocytic leukemia and small lymphocytic lymphoma, and hamper tumor progression both in patients and in tumor mouse models.^77^

### 4.4 OXPHOS pathway as a target

**FIGURE 3:**
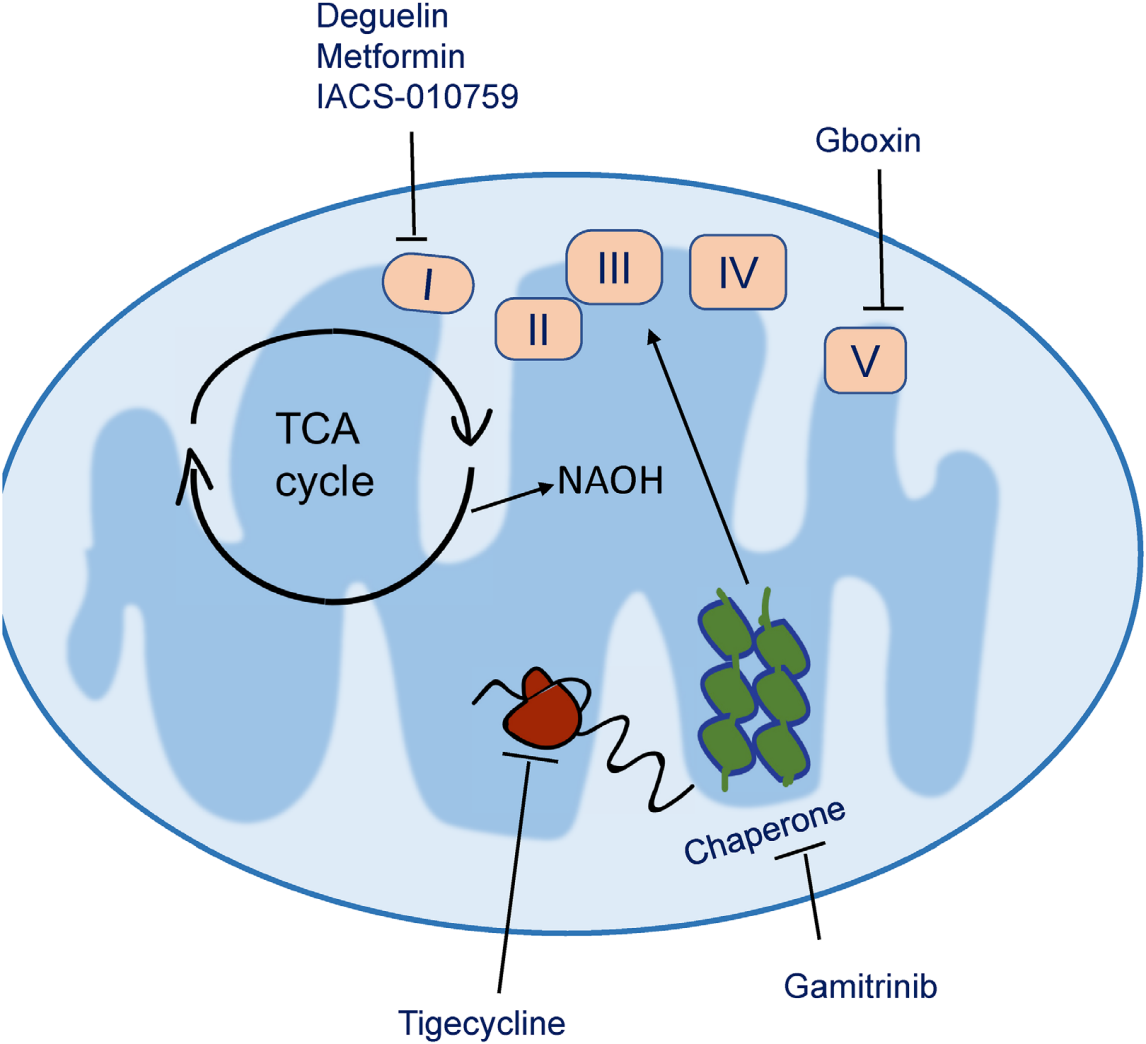
Molecule targeting the mitochondrial OXPHOS pathway. The OXPHOS pathway plays an essential role in mitochondria and tumorigenesis. Multiple small molecules have been developed to inhibit this pathway at several levels. Tigecycline^66^ inhibits translation of mitochondria mRNA into OXPHOS subunits; Gamitrinib^67^ inhibition OXPHOS assembly; Deguelin,^68^ metformin^69^ and IACS‐010759^70^ inhibits complex I in the OXPHOS pathway, while Gboxin^7^ inhibits complex V

The OXPHOS pathway couples with TCA and FAO and plays a central role in the mitochondria function (Figure 3). OXPHOS inhibition shows the beneficial effect in tumor progress both in mouse models and patients. The OXPHOS complex I inhibitor, metformin, is a widely used as antidiabetic medicine and has a recognized antitumor effect. An observational study published in 2005 suggested that the use of metformin was associated with a 23% decreased risk of any cancer type.^69^ After this, studies in many systems show the antitumor effect of metformin and its effect likely through inhibition of CSCs^78^ for the following reasons: (1) reduce the incidence of cancers; (2) reduce the malignancy; (3) reduce the likelihood of relapse. However, metformin is a weak OXPHOS inhibitor, its application in treatment of advanced tumors is still under investigation. On the other side, strong OXPHOS inhibitors (Rotenone, Oligomycin) have also been tested in multiple tumor models,^8^, ^79^ and these reagents show preferential inhibition of the stem‐like tumor cells. While the strong OXPHOS inhibitor have unacceptable side effects on normal cells, their applications in tumor treatments in patients are not very promising. A recent study in glioblastoma identified a novel OXPHOS inhibitor, Gboxin. Gboxin can recognize the unique feature of tumor cells and preferentially inhibit the OXPHOS pathway in tumor cells but not normal wild‐type cells. This might be a direction in future for developing antitumor reagents that target the mitochondria central pathway.^7^

### 4.5 Mitochondria and tumor microenvironment

The tumor microenvironment in solid tumors consists of extracellular matrix as well as the associated stromal cells including immune cells, fibroblasts, and vascular networks.^80^ Tumor cells are situated in highly heterogeneous microenvironments, both in cellular composition and metabolic profiling, the heterogeneity of oxygen distribution in tumor tissues leads to the heterogeneity of mitochondrial distribution. The hypoxia status inhibits the transcription and expression of many mitochondria genes encoded by nuclear, thus leads to the inhibition of mitochondrial biogenesis. The analysis of clinical data demonstrates the same. Thus, when using mitochondrial targeting reagents treat tumors, the effects of these reagents on tumor progression is a combined effect of these reagents on tumor cells as well as on the tumor microenvironment. This is a very intriguing area and requires further exploration.

### 4.6 Mitochondria and immunity

Mitochondrial and cellular metabolism is also critical for differentiation and effector functions of immune cells,^81^ Activated immune cells have high demands for ATP molecules for energy consumption and nutrients for anabolic synthesis to cater for their effector functions.^82^ For example, interfering of glycolysis or OXPHOS pathway disturbs the production of interferon‐γ of natural killer cells. Glycolysis is more essential for natural killer (NK) cell receptors–activated cell cytotoxicity since inhibition of glycolysis instead of OXPHOS decreased NK cell killing and attenuated NK cell degranulation and Fas ligand expression.^81^ The receptor‐interacting protein kinase 3 (RIPK3) plays an essential role in natural killer T (NKT) cell function via activation of the mitochondrial phosphatase phosphoglycerate mutase 5 (PGAM5). RIPK3‐mediated activation of PGAM5 promotes the expression of cytokines by facilitating nuclear translocation of nuclear factor of activated T‐cell (NFAT) and dephosphorylation of dynamin‐related protein 1 (Drp1), a GTPase is essential for mitochondrial homoeostasis.^83^ Thus, we always need to keep in mind the possible effect of using certain mitochondrial targeting reagent to treat tumors, especially for a tumor that is considered as an immunologically hot one.

## 5 CONCLUSION

It is now recognized that mitochondria, an organelle critical for biogenetics, biosynthesis, and many signaling, are reprogrammed by oncogenic pathways, oncogenic proteins or loss of tumor suppressors. Reprogrammed mitochondrial metabolism either is involved in initiating transformation of normal cells into tumor cells or provide tumor cell capabilities to survive in harsh microenvironment rending aggressive tumor growth. At the same time, these reprogrammed mitochondria might also reveal vulnerabilities of cancer cells and provide opportunity to develop drug specifically targeting mitochondria in cancer cells while leaving mitochondria in normal cells largely unaffected. Thus, exploring the mechanisms by which mitochondria is reprogrammed in each tumor setting and identifying corresponding vulnerability will be a promising direction for the next generation of antitumor drug development.
